# Multi-Parameter Physiological State Monitoring in Target Detection Under Real-World Settings

**DOI:** 10.3389/fnhum.2021.785562

**Published:** 2021-12-22

**Authors:** Yang Chang, Congying He, Bo-Yu Tsai, Li-Wei Ko

**Affiliations:** ^1^Institute of Bioinformatics and Systems Biology, National Yang Ming Chiao Tung University, Hsinchu, Taiwan; ^2^Center for Intelligent Drug Systems and Smart Bio-Devices (IDS2B), College of Biological Science and Technology, National Yang Ming Chiao Tung University, Hsinchu, Taiwan; ^3^Department of Electrical and Computer Engineering, National Yang Ming Chiao Tung University, Hsinchu, Taiwan; ^4^Drug Development and Value Creation Research Center, Kaohsiung Medical University, Kaohsiung City, Taiwan

**Keywords:** BCI, EEG, physiological state, attention, fatigue, stress

## Abstract

Mental state changes induced by stimuli under experimental settings or by daily events in real life affect task performance and are entwined with physical and mental health. In this study, we developed a physiological state indicator with five parameters that reflect the subject’s real-time physiological states based on online EEG signal processing. These five parameters are attention, fatigue, stress, and the brain activity shifts of the left and right hemispheres. We designed a target detection experiment modified by a cognitive attention network test for validating the effectiveness of the proposed indicator, as such conditions would better approximate a real chaotic environment. Results demonstrated that attention levels while performing the target detection task were significantly higher than during rest periods, but also exhibited a decay over time. In contrast, the fatigue level increased gradually and plateaued by the third rest period. Similar to attention levels, the stress level decreased as the experiment proceeded. These parameters are therefore shown to be highly correlated to different stages of the experiment, suggesting their usage as primary factors in passive brain-computer interfaces (BCI). In addition, the left and right brain activity indexes reveal the EEG neural modulations of the corresponding hemispheres, which set a feasible reference of activation for an active BCI control system, such as one executing motor imagery tasks. The proposed indicator is applicable to potential passive and active BCI applications for monitoring the subject’s physiological state change in real-time, along with providing a means of evaluating the associated signal quality to enhance the BCI performance.

## Introduction

Brain-computer interfaces (BCI) are methods that offer direct communication pathways between the human brain and external devices which have attracted much attention in various fields. They provide great potential for controlling machines, especially through a hands-free approach. To translate electroencephalographic (EEG) signals into actual commands they depend on users to generate EEG patterns recognizable to the system. These signals can be improved by appropriate training (Neuper and Pfurtscheller, 2009), however, even if with such training, the inevitable mental state changes of the individual during BCI usage still impact performance. Effects of attention and fatigue are regarded as the main causes of variation in the performance of BCIs (Myrden and Chau, 2015). Research has found that task engagement and attentional processes may impact the performance of several BCI techniques, such as P300 (Riccio et al., 2013) and motor imagery (MI) (Zhang et al., 2016). Mental fatigue produced by prolonged sequences of a cognitive task affects the accuracy of BCIs, as it decreases the separability of EEG signals (Talukdar et al., 2019). Furthermore, in a recent study by Zhang et al. (2020), it has been found that resting EEG modulation induced by stress is one of the key factors to signal-to-noise ratio and BCI performance. To address such impact of mental state changes on active BCIs, passive BCI has been proposed to assess the user’s cognitive state during ongoing BCI tasks, allowing the improvement in the interaction of human-machine system (Zander and Kothe, 2011).

On the other hand, people encounter various situations and events in daily life that elicit different emotions and physiological states than those in laboratory settings. Beyond their influences on BCI systems, some physiological states, such as stress and fatigue, are responsible for certain physical and mental health problems. For example, stress accounts for physical problems such as high blood sugar, high blood pressure, high cholesterol, obesity (Clark et al., 2011), and mental problems, e.g., depressive or anxiety disorders (Virtanen et al., 2007). Fatigue leads to decreased attention, which is the main cause of transportation accidents (Roets and Christiaens, 2019), and is reported to affect several psychological and physiological aspects of health (Lock et al., 2018). Besides stress and fatigue, other physiological states affect human life in various ways. Since physiological states play such important roles in both BCI and human life, how to measure and visualize them both within and beyond experimental settings is crucial and valuable.

A popular approach of deriving physiological states is from physiological signals, including EEG, electrocardiography (ECG) (Lampert, 2015; Hsu et al., 2017; Brás et al., 2018; Huang et al., 2018), electromyography (EMG) (Wijsman et al., 2013; Luijcks et al., 2014; Van Cutsem et al., 2017; Kehri et al., 2019), respiration (Widjaja et al., 2013; Grassmann et al., 2016), and galvanic skin response (GSR) (Villarejo et al., 2012; Fernandes et al., 2014; Krupinski et al., 2015; Kurniawan et al., 2016; Liu et al., 2016). Different physiological states regulate these autonomic physiological signals which naturally respond to changes in the central and peripheral nervous systems. Compared with other physiological approaches, EEG more directly reflects responses to the environment and external stimuli present in the processing of these events by the central nervous system, thus yielding more reliable outcomes. One of the most common methods for the investigation of brain dynamics is to identify the EEG spectral characteristics under varied conditions. Typically, EEG power spectra are quantified and divided by frequency ranges, such as delta (1–3 Hz), theta (4–7 Hz), alpha (8–12 Hz), beta (13–31 Hz), and gamma (32–50 Hz) bands (Tatum, 2014), with these oscillations within different frequency bands representative of various brain activations and conditions. Several publications in recent years have appeared documenting that EEG is a robust approach to evaluate physiological states, including alertness, drowsiness (Lin et al., 2010; Bajaj et al., 2020), fatigue (Monteiro et al., 2019; Xu et al., 2019), and stress (Xia et al., 2018; Asif et al., 2019). To assess the changes of physiological states exploiting EEG signals, five parameters are employed to develop the proposed pentagonal physiological state indicator: attention, stress, fatigue, and left and right brain activity levels.

Attention has been one of the most discussed cognitive functions in the field of neuroscience. It is an ability of an individual to selectively concentrate on relevant information while filtering irrelevant information. The attentional networks composed of alerting, orienting, and executive attention proposed by Posner and Petersen have been widely studied in terms of functional and anatomical perspectives (Posner and Petersen, 1990; Raz, 2004; Xuan et al., 2016). The attentional network test (ANT) is a behavioral task designed to evaluate the three stages of attention (Fan et al., 2002). The experiment that was developed to validate the proposed indicator also utilized ANT as a reference. Studies based on EEG indicate that attention can be estimated by detecting changes in alpha power activity, especially in the frontal lobe which is known to be responsible in regulating concentration (Quilodran et al., 2008; Doesburg et al., 2016). Research showed that the power spectra of theta and alpha increase when the subject is going into drowsiness (Jung et al., 1997; Lin et al., 2005); consistent experimental evidence has also demonstrated that mental engagement is inversely correlated to theta and alpha activities in the frontal region (Pope et al., 1995; Szafir and Mutlu, 2012). Our previous study has also demonstrated a real-time alertness detection system using this approach (Lin et al., 2010). Furthermore, research has applied similar approaches to determine attentional states during BCI experiments, providing a means to improve BCI performance (Lee et al., 2015).

Fatigue is a state that follows excessive physical or cognitive activity, thus it is typically partitioned as physical fatigue or mental fatigue (Jason et al., 2010). Physical fatigue is associated with body or muscle exhaustion, which leads to unsatisfactory physical performance. In contrast with physical fatigue, mental fatigue generally denotes a feeling of sleepiness or drowsiness, and it manifests as decreased attentional capacities and prolonged reaction times (Boksem et al., 2005), resulting in reduced efficiency. In a simulated driving experiment designed to study fatigue by Ma et al. (2019) subjects were allowed limited sleep and performed driving on tedious paths. It was found that alpha and beta activities in parietal and occipital lobes were strongly associated with the driver’s fatigue level (Ma et al., 2019). Another experiment exploiting simulated driving by Huang et al. (2016) demonstrated a closed-loop fatigue mitigation system that detected fatigue from the alteration of theta and alpha power in occipital components of recorded EEG. Similarly, experimental results of an ICA-based BCI study by Chuang et al. (2013) showed that increasing power in theta and alpha ranges were highly correlated with poor task performance and fatigue.

With the rapid pace of modern lifestyles, people race against time and experience different kinds of stressors. It is a well-known fact that stress causes negative effects on our health, emotions, and cognitive functions (McEwen and Sapolsky, 1995; Pearlin et al., 2005; Lazarus, 2006). However, mild-to-moderate stress has been found to be salutary, with this relationship between stress and performance described as the “inverted-U theory” (Sapolsky, 2015). The frontal lobes of the brain have been proposed as moderators of emotional regulation; extensive research has shown that frontal alpha asymmetry, representing the homologous differences between right and left frontal sites (typically F4–F3), of the is associated with affective processing (Harmon-Jones and Gable, 2018; Reznik and Allen, 2018). The asymmetry score is derived by subtracting natural log-transformed left hemisphere alpha power from the right, with positive asymmetry scores indicating stronger relative activity (Pfurtscheller et al., 1996). Specifically, it has been demonstrated that positive asymmetry scores are linked to positive emotions, whereas negative asymmetry scores are associated with stress responses and negative emotions (Meyer et al., 2014; Quaedflieg et al., 2015; Zhang et al., 2018a).

Motor imagery has been a widely discussed technique in BCI applications, and acts as an endogenous BCI, as it relies on spontaneous brain activities, as opposed to brain dynamics elicited by an external stimulus. The signals employed in MI-based BCI are based on principles of event-related desynchronizations and synchronizations, which correspond to changes of EEG power in the mu rhythm during either mental imagination of movement or actual motor execution (Pfurtscheller and Neuper, 1994; Pfurtscheller and Neurophysiology, 1997). Previous studies revealed electrode positions C3 and C4 are the most important ones for differentiating different motor imagery tasks by acquiring mu rhythm activities (Ramoser et al., 2000; Pfurtscheller et al., 2006).

To investigate the effectiveness of the proposed physiological state indicator, a prolonged experiment based on target detection was conducted. As acquiring EEG signals outside of experimental settings is impractical, a portable multi-channel EEG device was incorporated into the system development, as the long typical setup time and procedure needed for preparation of a standard off-the-shelf EEG system could affect the results in a study focusing on a time dependent effect like fatigue. In this study, a physiological state indicator with five parameters that reflect the physiological state changes based on EEG dynamics was developed, specifying the real-time fluctuations of the proposed parameters during the usage of BCI. These five parameters are attention, fatigue, stress, and the brain activities of the left and right hemispheres. The incorporated physiological states have been well-studied, with each having significant evidence for adopting specific EEG channels and frequency bands to derive them. The proposed experiment was designed to explore the neurophysiological changes using the indicator during a prolonged, attention-related task. With the proposed indicator, BCI usage can be evaluated according to a user’s physiological state changes, yielding improved BCI performances.

## Materials and Methods

**FIGURE 1:**
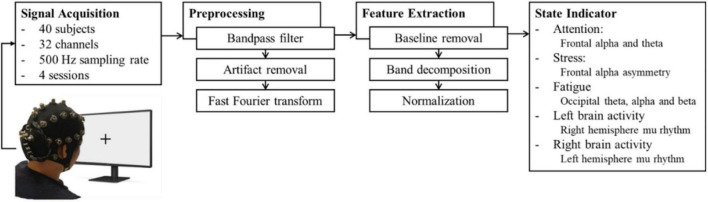
Signal processing for parameters. The EEG signals were acquired through a 32-channel wireless EEG device and preprocessed for artifact removal. Then, the signals of the particular sites of interest were decomposed and normalized to yield five parameters.

The parameters in the indicator were derived by EEG signal processing. We used portable wireless EEG equipment to acquire the EEG signals during the experiment to validate the effectiveness of the indicator. The signal processing flowchart is illustrated in Figure 1.

### Participants

Forty college students (30 males, 10 females) aged from 18 to 30 were recruited for participation in the study. No subjects reported histories of psychiatric or neurological disorders. All the participants were instructed to perform the task using their right hand. The experiments were administered at National Chaio Tung University (Hsinchu, Taiwan). The study was approved by the institutional review boards (IRB) of National Chaio Tung University.

### Experimental Paradigm

**FIGURE 2:**
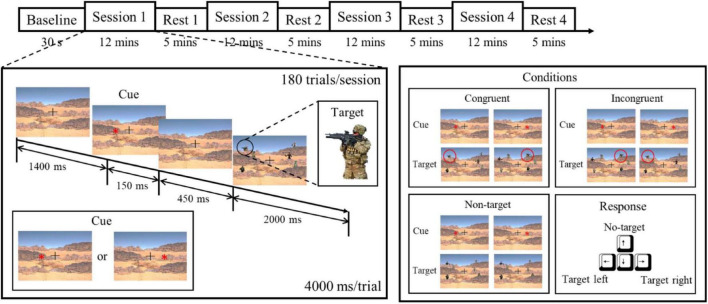
Experimental paradigm based on target detection. The experiment was composed of five sessions and five resting periods, with each session consisting of 180 trials. Each subject was instructed to respond to the target, and the conditions of the stimulus referred to the relationship between the cue and the target (He et al., 2021). To induce the effect of fatigue, the experiment lasted about 1 h.

In this study, we designed a target detection experiment that simulates a battlefield scenario to elicit state changes assessed by the proposed indicator (He et al., 2021). The experiment was composed of a 30-s EEG baseline recording and five blocks of 12-min sessions along with a 5-min rest session. First, the participant was seated in front of a 23.6” monitor with a relaxed posture. Then, the wireless EEG device was placed on the participant’s head and adjusted to fit. The participant was asked to fixate at the monitor showing a cross-mark for 30 s to establish a baseline recording. Afterward, the participant underwent a 68-min task to induce attention, fatigue, and stress level changes. In each trial, the participant was shown a cue representing in an asterisk on the left or right, indicating the probable location that a target would appear. The cue was followed by the presentation of a target, which was a figure of a soldier holding a rifle, accompanied with five other figures as distractors. The participants were required to press the button on the keyboard according to the direction of the target as soon as possible (Figure 2). Congruent conditions occurred if the direction of the target and the cue were the same and incongruent if they were opposite. To mimic more realistic circumstances, a condition with an absence of the target represented a no-target condition, for which the participant was required to press the “up” button. As the reaction time (RT) of the no-target condition is assumed to be longer, the criterion of maximal possible reaction time before a trial to be considered as fail is set at 2 s. Within a 12-min session, each of the three conditions were distributed between the 180 trials, resulting in 60 trials of each condition presented randomly during the session. Each session is followed by a 5-min rest. The total duration of the experiment was approximately 1 h, which was designed to bring about a mental state of fatigue. During the rest period, subjects were instructed to relax in order to ameliorate some of the neurological effects resulting from prolonged tension or discomfort produced during the active tasks; resting data from the period after the first 2 min of rest were used so as to minimize signal artifacts carrying over from the previous active stage. The combination of session and rest repeated four times, comprising the whole experiment. The attention parameter was assessed in both active and resting periods, while the fatigue was assessed and validated in the rest periods because other physiological state changes could interference the measurement of the fatigue parameter. To further investigate the effect of fatigue caused by continuous cognitive performance, the subjects were inquired to rate their self-evaluated fatigue level they perceived during task performances on a 10-point rating scale according to the method described by Karrer et al. (2004).

### Electroencephalographic Signal Acquisition and Preprocessing

To be compatible with the real-time state indicator, a wireless 32-channel EEG system called VEGA developed by Artise Biomedical, Taiwan was used for EEG acquisition. It consists of a Bluetooth module, miniature amplifiers, and semi-dry sponge-based electrodes with 32 designated channels: Fp1, Fp2, AF3, AF4, F7, F3, Fz, F4, F8, FT7, FC3, FCz, FC4, FT8, T7, C3, Cz, C4, T8, TP7, CP3, CPz, CP4, TP8, P7, P3, Pz, P4, P8, O1, Oz, and O2. The sponge electrodes used in the experiment function by absorbing saltwater prior to use; their design makes them flexible, adequate for hairy scalps, and usable for long-term EEG acquisition (Ko et al., 2019). The impedance of the skin-electrode interface was monitored constantly during the experiment. The references were located at the earlobes via two ear clips, and the sampling rate was set at 500 Hz. To obtain the features for physiological states, the acquired EEG signal was preprocessed for artifact removal and feature extraction. The signal underwent a 1–50 Hz bandpass filter for the preliminary removal of artifacts. The artifact removal from the EEG signal was carried out using the artifact subspace reconstruction (ASR) in EEGLAB toolbox, which is a component-based method capable of removing large-amplitude noise or artifacts in real-time, and is therefore practical for embedding in the software of portable devices (Chang et al., 2018). The ASR process comprises three steps: (1) Extracting reference, (2) determining threshold, (3) rejecting and reconstructing. To implement online ASR, the reference data were extracted using the baseline before the experiment onset. The reference data was applied to define the rejection threshold. Therefore, the ASR could reject artifact components and reconstruct cleaned data in near real-time using the established threshold (Chang et al., 2019). The signal was then processed via fast Fourier transform to obtain power spectral density (PSD) in decibel (dB). The PSD from the baseline was subtracted from the task periods. Finally, the signals went through band decomposition, yielding four commonly used frequency bands, delta (1–3 Hz), theta (4–7 Hz), alpha (8–12 Hz), and beta (13–31 Hz).

### Development of Physiological State Indicator

The features used to develop the five parameters were pre-defined, yet their scales were not specified *a priori*. To obtain easily understandable output parameters for each subject, the EEG power retrieved from 40 subjects was normalized to a usable scale. The general transformation formula was defined based on normalized t-score, but with the scale ranging from 0 to 10. The average power for each parameter was derived by averaging specific power across all subjects, as the power has been processed through baseline removal. Considering the existing and future outlier data, the normalization factor is modified to σ/2 instead of σ. Therefore, it enables 98.75% of the derived physiological state levels, i.e., *y*(*t*), to be distributed within 0–10 (μ *±* 2.5σ). Previous study has posited the feasibility of using this approach for normalization of EEG power values (Thatcher, 1998). The formula is defined as:


$$y\left(t\right)=5\pm \frac{1}{n}\sum_{C1}^{n}\frac{P_{C}\left(t\right)-\bar{P_{C}}}{f_{C}}$$

where *P* is the EEG band power of the region of interest (ROI), *c* is the channels included in ROI, *n* is the number of channels, and *f* is the normalization factor.

**FIGURE 3:**
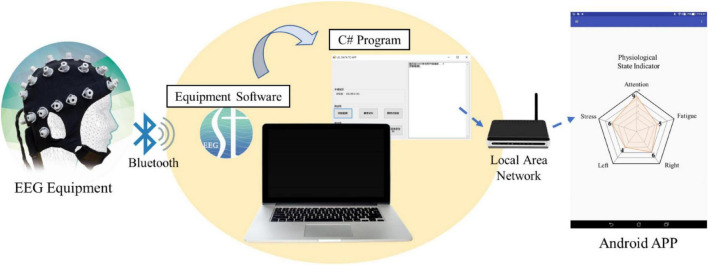
System information flow. The EEG acquisition is conducted with the software in the computer through Bluetooth. The acquired signal could be applied to a BCI system; at the same time, it is transmitted to the monitoring system running on portable devices. In the system, the signal is computed to easily assess the parameters which are displayed in a pentagon plot.

For a better, intuitive visualization, the magnitudes of the normalized parameters were displayed in a pentagonal indicator (Figure 3). Attention is known to be inversely proportional to the theta- and alpha-band amplitudes in the frontal region (Lin et al., 2010; Szafir and Mutlu, 2012), which were the F3, Fz, and F4 sites according to the channel locations on the EEG equipment. Therefore, the level of the attention parameter was defined as:


$$\text{Attention}\left(t\right)=5-\frac{1}{3}\left(\frac{P_{F3}\left(t\right)-\bar{P_{F3}}}{f_{F3}}+\frac{P_{Fz}\left(t\right)-\bar{P_{Fz}}}{f_{Fz}}+\frac{P_{F4}\left(t\right)-\bar{P_{F4}}}{f_{F4}}\right)$$

where *P* is the EEG power includes theta and alpha frequency bands.

As previous studies have reported, fatigue level is positively proportional to wide-band EEG power in the occipital region, including theta-, alpha-, and beta-band amplitudes, corresponding to the O1, Oz, and O2 electrodes (Chuang et al., 2013; Huang et al., 2016; Ma et al., 2019). The level of the fatigue parameter is defined as:


$$\text{Fatigue}\left(t\right)=5+\frac{1}{3}\left(\frac{P_{O1}\left(t\right)-\bar{P_{O1}}}{f_{O1}}+\frac{P_{Oz}\left(t\right)-\bar{P_{Oz}}}{f_{Oz}}+\frac{P_{O2}\left(t\right)-\bar{P_{O2}}}{f_{O2}}\right)$$

where *P* is the EEG power includes theta, alpha, and beta frequency bands.

Alpha asymmetry in the frontal region, measured F4/F3 signal ratios, was utilized as the stress parameter, defined as:


$$\text{Stress}\left(t\right)=5-\frac{P_{F4}\left(t\right)-P_{F3}\left(t\right)}{f_{C}}$$

where *P* is the natural log-transformed alpha power and *f_C_* is $\frac{std\left(P_{F4}-P_{F3}\right)}{2}$. Positive asymmetry scores represent stronger relative left than right activity as higher alpha band power reflects lower brain activity.

Similar to the parameters that positively correlate to the amplitudes of EEG power, the parameters of the left and right brain activity also followed a normalized formula. Hence, the parameter for left brain activity is defined as:


$$\text{Left}\left(t\right)=5+\frac{P_{C4}\left(t\right)-P_{Cz}\left(t\right)}{f_{C}}$$

and the parameter of right brain activity is defined as:


$$\text{Right}\left(t\right)=5+\frac{P_{C3}\left(t\right)-P_{Cz}\left(t\right)}{f_{C}}$$

where *P* is the EEG power within mu rhythm (8–13 Hz), *f_C_* is $\frac{std\left(P_{C4}-P_{Cz}\right)}{2}$ for left brain activation and $\frac{std\left(P_{C3}-P_{Cz}\right)}{2}$ for right brain activation, respectively.

Figure 3 depicts an informational flow diagram underlying the state indicator. While utilizing a BCI accompanied by the physiological state indicator, the acquired EEG signal was transmitted to the computer through Bluetooth. The user data would be recorded for 30 s to establish a baseline for removal, also as the reference data for ASR, as previously described. The recorded signal was then sent to a portable device by a computer program based on C# via local area networks with a refresh rate of 1 s. Any EEG state changes of the user during BCI usage would be converted into corresponding values of the proposed parameters based upon the mean and standard deviation of EEG power acquired by the 40-subject dataset. The indicator on the portable device displays the five parameters acquired from the connected computer on an easily accessible pentagonal plot in near real time.

## Results

### Behavioral Statistics

**FIGURE 4:**
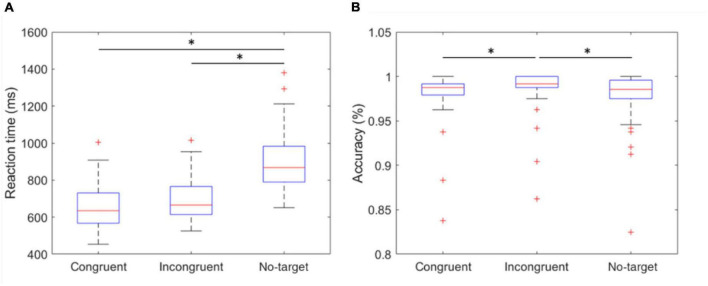
Reaction time and accuracy of congruent, incongruent, and no-target stimulus. **(A)** The mean RT of the three conditions. The RT of no-target stimulus was significantly longer than other conditions. **(B)** The accuracy of the three conditions. The incongruent stimulus induced the highest accuracy of 99.17% (IQR 98.75–100.00). *indicates that the difference between the two groups was statistically significant. The whiskers extend to the most extreme data points not considered outliers, and the outliers are plotted individually using the “+” marker symbol.

In the experiment, the stimuli are categorized as congruent, with the direction of the cue matching the target, incongruent, with the direction of the cue different from the target, or no-target, where the target is absent. To investigate the effects caused by different conditions, the RT and accuracy of the users’ responses were recorded. The condition of the congruent stimuli induced the shortest median RT of 634.7 ms (IQR 567.5–731.0). The incongruent stimuli yielded a slightly longer median RT of 665.7 ms (IQR 613.7–765.9). Other than the left and right stimulus correspondence choices, the subjects were required to press the “up” button after they recognized the absence of the target, which led to the longest median RT of 868.0 ms (IQR 789.5–983.0). The median accuracies of the congruent, incongruent, and no-target conditions were 98.75% (IQR 97.91–99.17), 99.17% (IQR 98.75–100.00), and 98.54% (IQR 97.50–99.58), respectively. The variables of RT and accuracy were compared using Kruskal-Wallis test, as they were not normally distributed. The *p*-values were adjusted for multiple comparisons using the Dunn and Sidák’s approach. A false discovery rate of *p* < 0.05 was considered statistically significant represented by an asterisk (*) (Figure 4).

### Validation of Parameters

**FIGURE 5:**
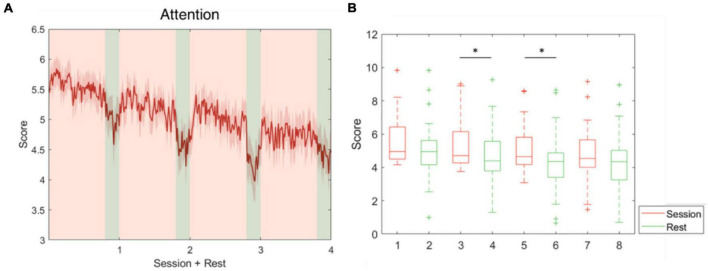
Attention level fluctuations during the whole experiment. This parameter was developed using theta and alpha-band power at F3, Fz, and F4. **(A)** The average attention level across the four session and rest periods. **(B)** The attention level during the sessions decreased over time and was significantly higher than during the following rest periods in pairs 2 and 3. *indicates that the difference between the two groups was statistically significant. The whiskers extend to the most extreme data points not considered outliers, and the outliers are plotted individually using the “+” marker symbol.

**FIGURE 6:**
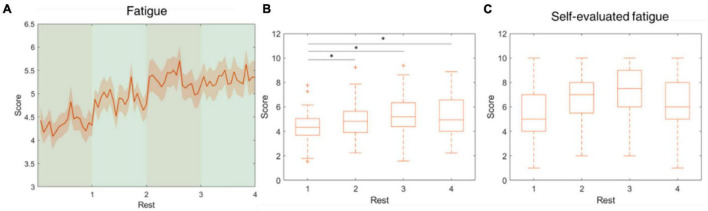
Fatigue level fluctuations during the rest periods. This parameter was developed using theta, alpha and beta-band power at O1, Oz, and O2. **(A)** The average fatigue level across the four rest periods. **(B)** The fatigue level increased from rest 1 to rest 3 and mitigated in rest 4. **(C)** The self-evaluated fatigue level over four rest periods. *indicates that the difference between the two groups was statistically significant. The whiskers extend to the most extreme data points not considered outliers, and the outliers are plotted individually using the “+” marker symbol.

**FIGURE 7:**
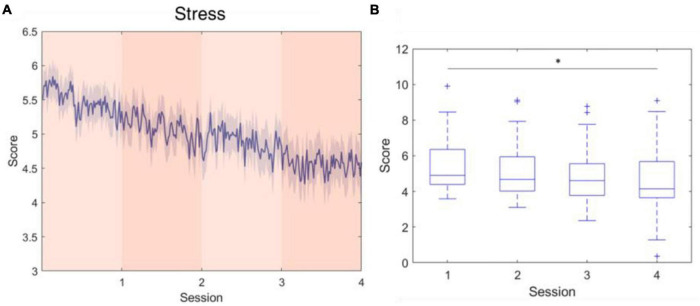
Stress level fluctuations during the active sessions. This parameter was developed using log-transformed alpha-band power at F3 and F4. **(A)** The average stress level across the four session periods. **(B)** The stress level significantly decreased over time. *indicates that the difference between the two groups was statistically significant. The whiskers extend to the most extreme data points not considered outliers, and the outliers are plotted individually using the “+” marker symbol.

**FIGURE 8:**
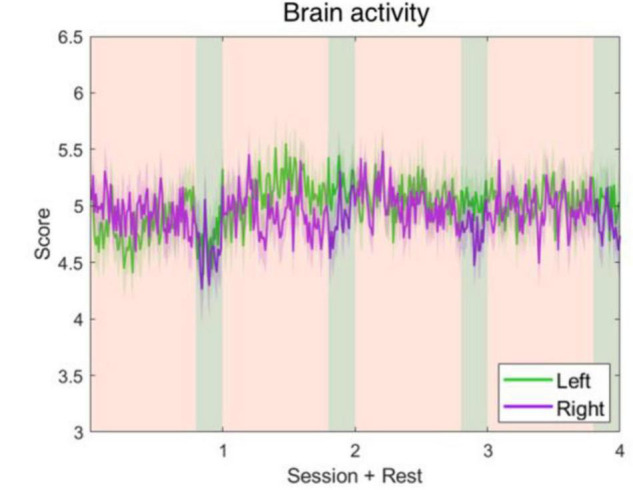
Left and right brain activity level fluctuations during the whole experiment. The parameters were developed using alpha-band power of C4–Cz and C3–Cz. It shows that the left and right brain activities are approximate to each other, and neither ascended nor descended significantly.

The five parameters within the proposed physiological state indicator were developed using particular combinations of brain regions and EEG frequency bands. Each parameter, respectively, represents a distinct mental state, therefore, we utilized different periods in the experiment for validation. To assess the attention parameter, the whole experiment was included to obtain the dynamics of attention throughout the four experimental sessions and rests. The results demonstrated that attention levels during the active sessions were significantly higher than their subsequent rest periods, but also exhibited a decay over time (Figure 5). The attention level during the rest periods also declined over time and eventually stabilized by the final period. The rest periods in the experiment were used to study the fatigue levels affected by the long sessions during which the subjects were highly focused. The experiment was intentionally lengthy, reaching nearly 1 h, with the intention of inducing fatigue. Consequently, the fatigue levels increased gradually and plateaued by the third rest period (Figure 6). In addition to the assessment by EEG signals, self-evaluated fatigue levels were recorded for comparison (Figure 6C). The stress parameter was intended to respond to the stress that the users experience across different sessions. It illustrated that the stress levels were at their highest in the beginning of the experiment, and then significantly decreased over the four session periods (Figure 7). Since the participants were instructed to perform the experiment with their right hand, hypothetically the derived right brain activity levels should be higher than the left brain activity level during all session periods and approximate to the left brain activity level in all rest periods. However, the results revealed that the left and right brain activities were close to each other, and did not appear to exhibit significant trends (Figure 8). The levels of the parameters of each subject were averaged per period for statistical analysis, yielding one average value of the physiological state level per subject in one period (session or rest). The comparable distributions passed the Kolmogorov-Smirnov test for normality. The average levels of the attention parameter between each pair of session and rest were compared using the paired *t*-test. The average levels of the fatigue and stress parameters were compared using the repeated measure ANOVA with *post-hoc* test using Dunn and Sidák’s approach. The alpha level of 0.05 was used for statistical hypothesis testing.

## Discussion

In this study, we developed a physiological state indicator integrated with wireless EEG equipment, enabling the assessment of physiological states for potential passive and active BCI applications. Prior active BCI studies have documented the effects of physiological state changes on BCI performance (Riccio et al., 2013; Myrden and Chau, 2015; Talukdar et al., 2019; Zhang et al., 2020). During long-term BCI performance users’ physiological states change spontaneously, which lead to unsatisfactory outcomes. However, passive BCIs may facilitate the use of traditional, active BCIs by providing an estimation of the underlying user state variations (Millán et al., 2010). Parameters that were reported to affect BCI performance, including attention, fatigue, and stress were adopted in the proposed system, and the left and right brain activity parameters were designed specifically for MI-based BCI, yielding a collection of five parameters for BCI assessment. The experiment based on ANT not only induced attentional alterations, the prolonged duration also caused fatigue in the subjects and the moderate difficulty allowed subjects to adapt, producing transitions in stress levels.

To induce attention differences between active sessions and rest periods, but not produce excessive fatigue over the 1-h duration, we designed an experiment that was based on target detection and ANT, somewhat modified to be closer to real-world scenarios. With this experiment, the relationship among these three conditions and the physiological state changes of the users can be evaluated. Generally, incongruent conditions introduce “conflict” that requires longer information processing times, resulting in longer RTs. In our experiment, the target in the no-target condition is absent, hypothetically causing even longer RTs than the incongruent conditions. The no-target condition demonstrated a lower accuracy of 98.54% and a significantly longer median RT of 868.0 ms, compared to the congruent and incongruent conditions. The conflict in the incongruent condition did bring about slightly longer median RT, however, the had the highest accuracy among the conditions. This may have been caused by the relatively simple experimental design compared to similar cognitive experiments, such as the classical ANT protocol. As the result, the median accuracies for all conditions exceeded 98%. Nevertheless, the experimental procedure provided the necessary factors to elicit physiological state changes, especially for the adopted parameters. Although the participants were instructed to respond as soon as possible, the maximal possible reaction time before a trial was considered as failed was set at 2 s because the experiment included the no-target condition. This was assumed to require a longer reaction time, resulting a ceiling effect for performance. However, several participants performed relatively poorly, and there were significant differences between congruent/incongruent and incongruent/no-target conditions, indicating that the three conditions could elicit the variations in performance within participants, despite the ceiling effect.

A large body of prior literature has indicated that neurophysiological signals contain information related to physiological state changes, which were acquired via EEG in our experiment (Lin et al., 2010; Xia et al., 2018; Asif et al., 2019; Monteiro et al., 2019; Xu et al., 2019; Bajaj et al., 2020). Some of the research in recent years has applied machine learning or deep learning methods to identify different stages of physiological states (Jebelli et al., 2019; Ma et al., 2019; Khessiba et al., 2021). However, the high computational power that deep learning demands does not necessarily provide better classification performance due to inter-subject variabilities. For example, in studies that investigated human fatigue during driving simulation experiments, one experiment deployed an EEG classifier based on convolutional neural networks (CNN), which demonstrated an average accuracy of 92.68% in the intra-subject test (Zeng et al., 2018). By contrast, it demonstrated a reduced performance of 84.38% in an inter-subject binary classification, falling beneath the performance reported by Ko et al. (2015), which employed a linear regression model on a 1–30 Hz EEG power spectrum array. A review study by Monteiro et al. (2019) also demonstrated that linear models like fisher linear discriminant analysis (FLDA) could outperform deep belief network (DBN) while using PSD to assess mental fatigue in cross-subject classification. In consideration of the computational capacity necessary to process five parameters in real-time on portable devices, we implemented models that determine physiological state levels using linear transforms of EEG power amplitudes. Additionally, adaptive BCIs have been proposed to deal with the ubiquitous non-stationarities in EEG signals (Shenoy et al., 2006). Linear transformation models like our indicator benefit the such adaptive models by providing consecutive levels that correspond to user physiological state changes rather than binary classification (Krol et al., 2016; Zhang et al., 2018b).

To study and validate the indicator that was developed based on cognitive state changes, a prolonged experiment incorporating EEG signal recording was administered. In general, long-term task performance results in increases in fatigue and reductions in goal-directed attention (Boksem et al., 2005). In the validation of the attention parameter, the attention levels in the active sessions surpassed those in the subsequent rest periods, and fluctuations indicated that the users’ mental engagements dropped in the resting periods, then ascended right after the task onset. In addition, attention level exhibited a decay over time in both active sessions and rest periods, suggesting that attention reduced throughout the experiment. In other words, the subject’s theta and alpha band amplitudes in the frontal region increased over time under both conditions. However, it is generally accepted that attention is categorized into a variety of types based upon functional aspects. Sustained attention represents the attentional functionalities that determine the cognitive capacity and efficacy of selective and divided attention (Sarter et al., 2001), which is represented by the attention parameter, and reflects the vigilance, of an individual during long-term BCI usages.

The cognitive demands during the experiment cause mental fatigue, which is assumed to increase over time. Trejo et al. (2015) demonstrated that the EEG signs of developing mental fatigue are present 15–30 min after task onset. In our paradigm, physiological state changes other than fatigue could confound the measurement of the fatigue parameter. Therefore, the fatigue was assessed and validated in the rest periods, as opposed to the attention parameter which was assessed in both active and resting periods. The results showed that the estimated fatigue levels during the rest periods, which followed 12-min cognitive performance sessions, progressively ascended and peaked in the third period, indicating the subjects were experiencing maximal fatigue by that time. Correspondingly, the self-evaluated fatigue level in period 3 reached an average of 7.25 points out of 10, which was also the highest among the four rest periods. The fatigue level demonstrated a similar trend to the cluster of “strugglers” proposed by Karrer et al. (2004), which presented a high level of fatigue during a simulated driving test and subsequent reduction of fatigue toward the end of the driving test. We inferred that the perception of the impending end of the experiment resulted in a reduction of fatigue level, possibly indicating a mental relaxation in the subject.

Stress is a parameter that was not specifically elicited in the experimental design, instead, the spontaneous responses of the subjects during four sessions were recorded. Compared with the EEG characteristics that correlate to attention and fatigue, frontal alpha asymmetry is a relatively well-established electrophysiological feature for stress assessment. In a study proposed by Arpaia et al. (2020), frontal alpha asymmetry was applied to machine learning classifiers and achieved more than a 90% accuracy in classifying 2-s EEG epochs using a wearable EEG instrument. Similarly, the F4/F3 asymmetry was adopted in this study to assess the stress that the subject experienced in the successive performance of the cognitive task. As the experiment proceeded, subjects’ stress levels exhibited a descending trend, indicating that the subjects felt less anxious toward the last session, according to gradually increased F4-F3 asymmetry scores. Generally speaking, situations that are unpredictable, ambiguous, or unfamiliar are more likely to elicit stress (Michie and medicine, 2002). In our experiment, which did not include induced stressors, the unfamiliarity of the first session, where the subjects experienced the highest level of stress, was posited as the main factor of stress; this elevated stress level was mitigated as the subjects habituated the experiment.

One limitation of the proposed indicator is that the advanced algorithms of MI-BCIs were not included in the parameters of left and right activity. The MI-BCI algorithms have been extensively studied to distinguish the EEG patterns representative of the mental imagination of different movements. Simply measuring the mu rhythm power of the left and right motor cortices might not be sufficient to differentiate motor executions based on the results of the left and right brain activity parameters. Estimating the subject’s event-related desynchronization/synchronization is fundamental to MI, however, additional algorithms utilizing the existing parameters are required for accurate MI-BCI control.

In this study, the features for the five parameters were adopted based on online signal processing using the findings of previous studies. The experiment intended to elicit the variations in the parameters of the subjects, specify the EEG power ranges of the parameters, and thus develop a real-time multi-parameter physiological state indicator with proper scaling. In the experimental design, the general attention and fatigue were assessed, while the stress, left activity, and right activity were studied through the spontaneous responses of the participants. However, to investigate each of the parameters throughout, sophisticated experimental design for each of the parameters would benefit the investigation of the physiological state changes. Furthermore, the parameters have intricate interactions and are not independent mechanisms. For instance, the impact of fatigue on attention (Boksem et al., 2005; Faber et al., 2012), the interactions between attention and stress (Chajut and Algom, 2003; Zhang et al., 2020), and even the effects of fatigue and stress on MI (Talukdar et al., 2019; Schlatter et al., 2020). Such usage of a general indicator doesn’t warrant a detailed intra-parametric analysis, though that could be an interesting direction for generating a future indicator.

Mental workload is a parameter we would like to investigate and incorporate into the system in the future. Despite different causes, the physiological correlates of mental workload and stress manifest in rather similar ways (Parent et al., 2019). Likewise, research by Käthner et al. (2014) reported that a high mental workload, as well as fatigue, affects event-related potential (ERP) -based BCI performance. The results of our previous work reported in Zhang et al. (2020) also indicated that stress-dependent effects are similar to the outcome of fatigue, leading to a potential conclusion that stress and fatigue both impact attention capacity and interact to affect BCI performance.

All in all, future experiments should be designed and managed carefully as the causes and consequences of the physiological states including mental workload, attention, stress, and fatigue can be easily confounded. It is worth mentioning that currently the values of the parameters in the indicator are derived from the 40-subject EEG data. The indicator software on portable devices can instantly reflect the physiological states of the user based on the existing dataset. In addition, with accurate labeling of corresponding physiological states, parameters in future indicator can benefit from the acquired EEG signals during potential experiments, as the collected data provide more neurophysiological information for parameter calibration. As such, the dataset would be expanded and bring about more generalized results.

**FIGURE 9:**
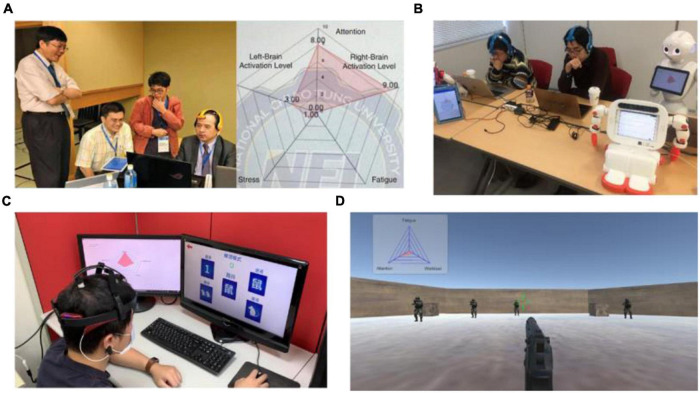
Real-world applications of the proposed physiological states indicator. **(A)** A Go player using the system while playing Go with smart machines for machine-human co-learning. **(B)** Intelligent agent for robotic edutainment in mathematical and language learning. **(C)** Game-based assessment of cognitive functions. **(D)** Training of soldiers using first person shooting scenario.

With the increasing quality of commercial EEG devices and advancements in artifact removal techniques, using BCIs in parallel with physiological state assessment tools in daily living has become more realistic. The validation results suggest that the neurophysiological manifestations of state changes are consistent across a variety of cognitive processes, setting the stage for practical applications of mental state indicators in passive BCIs. The proposed multi-parameter indicator has since been applied to a variety of fields in real-world environments to strengthen the practicability of the system. In 2018, a study adopted the physiological state indicator to aid professional and amateur Go players in competing with smart machines (Lee et al., 2020; Figure 9A). The wearable BCI system benefits machine-human co-learning as it can be integrated with training methods or neurofeedback to monitor and regulate Go players’ physiological states, helping them to maintain their attention and overcome the competitive pressure experienced in the game. Later, the system was extended to the educational domain interfacing with an intelligent agent for robotic edutainment in 2019 (Lee et al., 2019; Figure 9B). The experimental results showed that through playing games, the system improved the interest and performance of students in mathematical and language learning. Currently, the proposed indicator is also being applied to a game-based assessment for cognitive function in Kaohsiung Medical Hospital (Figure 9C) and a training paradigm for military soldiers using a first person shooting scenario in National Chung Shan Institute of Science and Technology (Figure 9D). To sum up, by estimating physiological state changes precisely using artifact-free EEG signals, the indicator can advance applications in interdisciplinary fields.

## Conclusion

To our knowledge, this is the first study presenting a system that indicates easily accessible metrics regarding multiple physiological states via wireless EEG in real-time. While BCI has been demonstrating great potential for controlling external devices with our minds, the physiological state changes induced by excessive stimulus under experimental settings or real conditions not only impact task performance and BCI accuracy, but are also interconnected with physical and mental health. Therefore, the concept of a passive BCI indicator is proposed to address such conditions by identifying the users’ spontaneous brain activities in real time. In this study, the parameters integrated into the state indicator include attention, fatigue, stress, and left and right brain activities, which were derived according to the cognitive state changes gathered from EEG signal processing. Furthermore, a prolonged experiment based on target detection was developed to validate the proposed indicator. The experimental results demonstrated a feasible outcome that corresponded to different stages during the experiment. The proposed physiological state indicator is applicable to online BCI applications, simultaneously allowing the state of the user to be assessed, along with providing a means of continuously evaluating the associated control signal quality.

## Data Availability Statement

The raw data supporting the conclusions of this article will be made available by the authors, without undue reservation.

## Ethics Statement

The studies involving human participants were reviewed and approved by the Institutional Review Boards of National Chaio Tung University. The patients/participants provided their written informed consent to participate in this study.

## Author Contributions

L-WK initialized the research idea, designed the experimental paradigm, and advised the methods of EEG data analysis. L-WK, YC, and CH performed the data analysis of EEG signals and drafted the manuscript. CH and B-YT performed the EEG data collection and provided advice on the manuscript revisions. L-WK, YC, and B-YT developed the software of the physiological state indicator. All authors agreed on the final manuscript submission.

## Conflict of Interest

The authors declare that the research was conducted in the absence of any commercial or financial relationships that could be construed as a potential conflict of interest.

## Publisher’s Note

All claims expressed in this article are solely those of the authors and do not necessarily represent those of their affiliated organizations, or those of the publisher, the editors and the reviewers. Any product that may be evaluated in this article, or claim that may be made by its manufacturer, is not guaranteed or endorsed by the publisher.
